# Role of post-translational modifications of Sp1 in cardiovascular diseases

**DOI:** 10.3389/fcell.2024.1453901

**Published:** 2024-08-26

**Authors:** Xutao Sun, Qi Zhou, Chengpu Xiao, Caiyun Mao, Ying Liu, Guozhen Chen, Yunjia Song

**Affiliations:** ^1^ Department of Synopsis of the Golden Chamber, Heilongjiang University of Chinese Medicine, Harbin, China; ^2^ Department of Pharmacology, Heilongjiang University of Chinese Medicine, Harbin, China; ^3^ Department of Typhoid, Heilongjiang University of Chinese Medicine, Harbin, China; ^4^ The Second Affiliated Hospital of Hunan University of Traditional Chinese Medicine, Changsha, Hunan, China; ^5^ Department of Pediatrics, Yantai Yuhuangding Hospital, Shandong, China

**Keywords:** Sp1, post-translational modification, cardiovascular diseases, cell signaling, biomarkers

## Abstract

Specific protein 1 (Sp1) is pivotal in sustaining baseline transcription as well as modulating cell signaling pathways and transcription factors activity. Through interactions with various proteins, especially transcription factors, Sp1 controls the expression of target genes, influencing numerous biological processes. Numerous studies have confirmed Sp1’s significant regulatory role in the pathogenesis of cardiovascular disorders. Post-translational modifications (PTMs) of Sp1, such as phosphorylation, ubiquitination, acetylation, glycosylation, SUMOylation, and S-sulfhydration, can enhance or modify its transcriptional activity and DNA-binding stability. These modifications also regulate Sp1 expression across different cell types. Sp1 is crucial in regulating non-coding gene expression and the activity of proteins in response to pathophysiological stimuli. Understanding Sp1 PTMs advances our knowledge of cell signaling pathways in controlling Sp1 stability during cardiovascular disease onset and progression. It also aids in identifying novel pharmaceutical targets and biomarkers essential for preventing and managing cardiovascular diseases.

## 1 Introduction

The mammalian RNA polymerase II transcription factor Sp1 (specific protein 1) was initially identified and is implicated in numerous critical biological processes (Yu et al., 2021). It contains three highly homologous Cys2-His2 regions in its DNA-binding domain, promoting gene transcription. Sp1 regulates physiological processes such as angiogenesis, inflammation, lipid metabolism, endothelial dysfunction, pathological cardiac hypertrophy, hypertension, and aortic aneurysm. These processes include inflammation (Dunzendorfer et al., 2004; Bertocchi et al., 2011; Singh et al., 2016), angiogenesis (Lin et al., 2011; Lu et al., 2023a), plaque stability (Kavurma et al., 2001; Li et al., 2019), endothelial dysfunction (Singh et al., 2016; Lu et al., 2023a; Saha et al., 2016), lipid metabolism (Yang et al., 2016; Ochiai et al., 2016; Kuang et al., 2017; Okoro et al., 2015; Hughes et al., 2002), and vascular smooth muscle cells (VSMCs) apoptosis (Zhang et al., 2021). Sp1’s involvement in various cellular processes is underscored by its interactions with key regulators and proteins such as Angiotensinogen II, Pai-1, mitochondrial calcium uptake protein 1 (MICU1), transforming growth factor alpha (TGFA), and Von Willebrand factor (vWF). Post-translational modifications (PTMs) are crucial for the function of intracellular regulatory proteins like Sp1 (Jiang et al., 2022).

Phosphorylation, deubiquitination, acetylation, glycosylation, SUMOylation, and S-sulfhydrylation can either enhance or diminish Sp1 protein stability, affecting its binding efficiency to downstream components and the progression of the cell cycle. Thus, a thorough investigation into the PTMs of Sp1 and their impact on the development and advancement of cardiovascular diseases offers a fresh perspective on the molecular mechanisms underlying these conditions. It aids in creating novel therapeutic targets and biomarkers, essential for preventing and managing cardiovascular diseases. This article discusses the molecular makeup, physiological role, and PTMs of Sp1 in controlling cardiovascular disease.

## 2 Structure and function of Sp1 protein

### 2.1 Structure of Sp1

**FIGURE 1:**
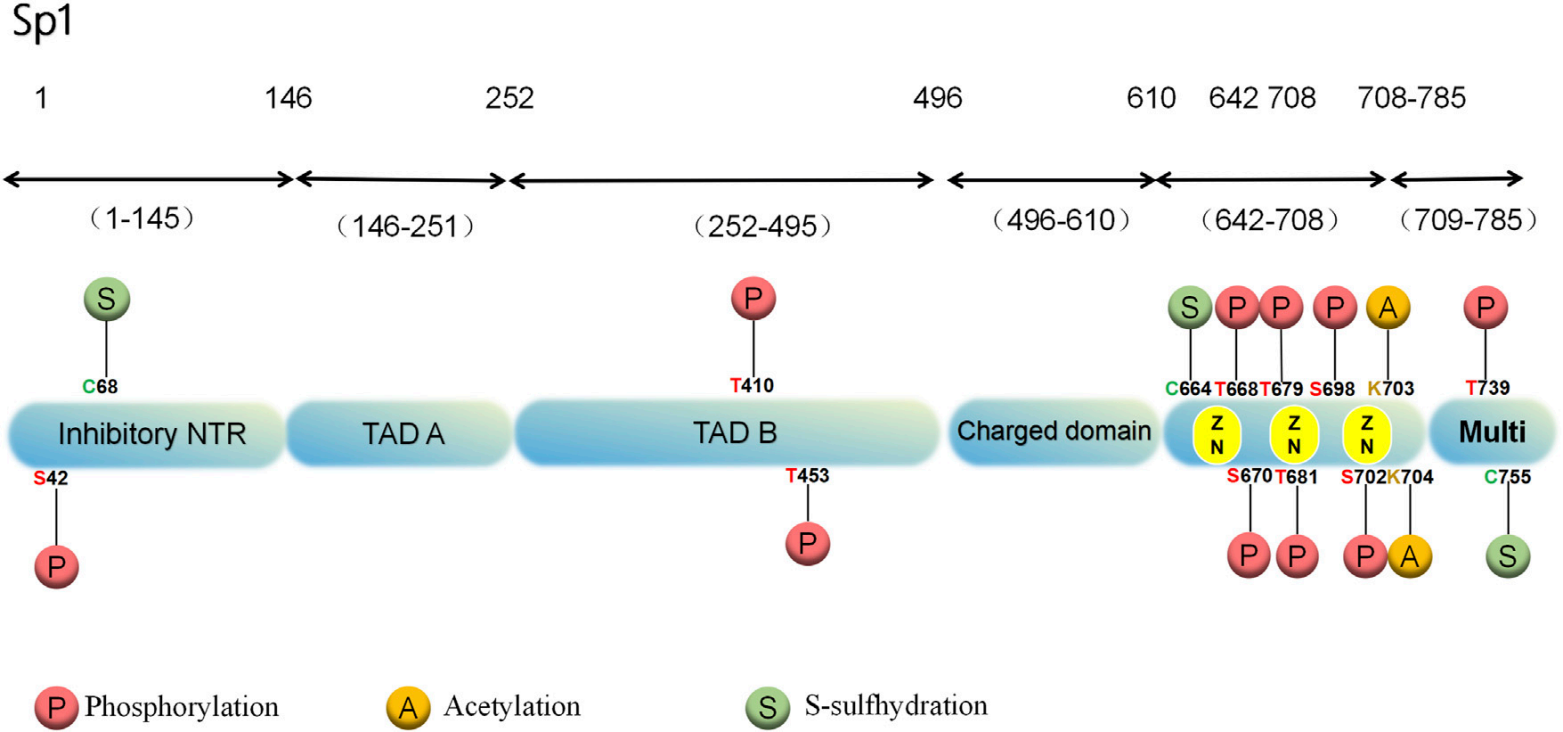
Structure and PTMs sites associated with cardiovascular diseases of the human Sp1 protein.

A greater abundance of Sp1 is found in cells’ nuclei than in the cytoplasm. It has low tissue specificity and is distributed on many organs, including brain, kidney, lymph and bone marrow (Yu et al., 2021). According to human chromosome linkage map analysis, Sp1 binds to a minimum of 12,000 sites (Jiang et al., 2022). In structure, the Sp1 protein contains 785 amino acid residues and an 81 kDa molecular weight with 164 serine and threonine residues (Yu et al., 2021). Various domains are involved, such as the N-terminal repression domain, glucose- and serine/trihydrogen-abundant domain, DNA-binding domain of zinc-finger and C-terminal (Lee et al., 2005; Courey and Tjian, 1988) (Figure 1). In Sp1, glutamine-abundant transcriptional stimulation regions, called A and B, target a variety of PTMs (Jiang et al., 2022). The active center of Sp1 is made up of three highly similar Cys2-His2 regions located in the C-terminal DNA-binding region of Sp1, which interacts with GC-rich DNA sequences to enhance gene transcription (Yu et al., 2021; Jackson et al., 1993). The DNA-binding region of Sp1 is highly conservative, but the N-terminal region of its protein is more differentiated (Kaczynski et al., 2003).

### 2.2 Function of Sp1 protein and cardiovascular disease

Sp1 has been extensively studied for its involvement in regulating numerous genes, including steward genes and active regulatory genes (Yu et al., 2021). The Sp1 protein regulates a variety of processes in cells, including differentiation, proliferation, migration, invasion, apoptosis and DNA repair (Yang et al., 2016). More importantly, Sp1 has also been shown to be associated with the occurrence and progression of a variety of cardiovascular system diseases. Specifically, first, Sp1 can be involved in lipid metabolism through transcriptional regulation. It can not only affect the cholesterol levels by regulating the influx, esterification and efflux of cholesterol (Kuang et al., 2017; Okoro et al., 2015; Saleheen et al., 2015; Chen et al., 2011), but also regulate the lipoprotein levels in the blood by inhibiting the gene transcription of lipoprotein lipase (LPL) with hydrolytic triglycerides (Hughes et al., 2002; Harris et al., 2008); Next, Sp1 regulates the plaque stability (Bertocchi et al., 2011; Singh et al., 2016; Kavurma et al., 2001; Li et al., 2019; Lin et al., 1997); In addition, Sp1 can bidirectional regulate the proliferation, migration, and phenotypic conversion of vascular smooth muscle cells (VSMCs) (Zhang et al., 2021; Bonello and Khachigian, 2004; Imayama et al., 2008; Tan et al., 2008; Sugawara et al., 2001; Wang et al., 2006; Chan et al., 2010) Furthermore, the activation of Sp1 has been found to be associated with the proliferation, migration, and senescence of vascular endothelial cells (VECs), and thus plays a certain regulatory role in blood mobility, fibrinolysis, vascular tension and platelet aggregation (Lin et al., 2011; Saha et al., 2016; Xu et al., 2004; Reisinger et al., 2003; Bai and Chen, 2021; Pagès and Pouysségur, 2005; Hasegawa et al., 2006); Additionally, Sp1 has the ability to modulate chronic inflammation within the walls of blood vessels (Dunzendorfer et al., 2004; Bertocchi et al., 2011; Singh et al., 2016).

Post-translational modifications (PTMs) such as phosphorylation, acetylation, ubiquitination, O-GlcNacylation, and SUMOylation have been shown to influence the function and stability of Sp1. These PTMs of Sp1 play a crucial role in regulating the function of the proteins it encodes as well as the regulation of non-coding genes. The impact of Sp1 PTMs on cardiovascular disease is further explored in the subsequent discussion, particularly in relation to the regulation of Sp1 stability and transcriptional activity.

## 3 The regulatory effect of PTMs of Sp1 protein on cardiovascular diseases

### 3.1 Sp1 phosphorylation and cardiovascular diseases

Sp1’s significant role in gene regulation has garnered extensive interest and investigation. Recent evidence suggests that Sp1’s phosphorylated state-especially notable in cardiovascular disease research-may be crucial in regulating several genes (Chu and Ferro, 2005). Phosphorylation, a major PTM, involves the addition of phosphoryl groups primarily to serine, threonine, as well as tyrosine sites on proteins through the action of kinases (Wang and Wang, 2019). Numerous kinases, including Akt (Kavurma et al., 2001; Tan and Khachigian, 2009), casein kinase 2 (CK2) (Dunzendorfer et al., 2004; Bertocchi et al., 2011; Li et al., 2015), extracellular signal-regulated kinase (ERK) (Yang et al., 2016; Ochiai et al., 2016; Kaczynski et al., 2003; Bonello and Khachigian, 2004; Xu et al., 2004; Imayama et al., 2008), p38 kinase (Lin et al., 2011), and protein kinase C zeta (PKC-ζ), as well as other kinases identified by molecular weight and activity, phosphorylate Sp1 *in vitro*. These kinases either enhance or reduce Sp1’s transcriptional activity.

#### 3.1.1 Sp1 phosphorylation and atherosclerotic plaque

Tissue factor (TF), a glycoprotein found in cell membranes, plays a role in the catalytic conversion of prothrombin to thrombin, with elevated expression in atherosclerotic plaques (Grover and Mackman, 2020; Bozkaya et al., 2023). Lin et al. (1997) discovered that shear stress stimulates TF gene expression, phosphorylates Sp1, and enhances its transcriptional activity, thereby contributing to the development of atherosclerotic plaques. This effect is comparable to the influence of the phosphatase inhibitor okadaic acid (OKA).

Collagen in atherosclerotic plaques is a predominant constituent of the extracellular matrix, which is controlled by prolyl-4-hydroxylase α1 (P4Ha1) (Li et al., 2019; Katsuda and Kaji, 2003). Li et al. (2019) treated MOVAS cells, a mouse smooth muscle cell (SMC) line, with melatonin and demonstrated that melatonin regulated P4Ha1 expression through the Akt/Sp1 signaling pathway. Furthermore, high-dose melatonin therapy enhanced the durability of atherosclerotic plaques in ApoE−/− mice through increasing AKT-mediated phosphorylation of Sp1 and promoting Sp1’s attachment to the P4Ha1 promoter, thereby increasing P4Ha1 expression in SMCs (Li et al., 2019). However, VSMCs apoptosis has been found to destabilize atherosclerotic plaques, leading to rupture, thrombosis, and sudden death (Bennett et al., 2016). Kavurma et al. (2001) reported that Sp1 phosphorylation is a prerequisite for inducing VSMCs apoptosis. CAM, an inhibitor of DNA topoisomerase I, induces various types of apoptosis (Reisinger et al., 2003). When CAM is introduced to atherosclerotic VSMCs, it phosphorylates Sp1, triggering the transcription of the pro-apoptotic gene FasL in VSMCs. This, in turn, promotes apoptosis in VSMCs and reduces the stability of atherosclerotic plaques (Kavurma et al., 2001).

Toll-like receptor 2 (TLR2), a functional inflammation-related receptor, promotes the formation of atherosclerosis (Singh et al., 2016; Mullick et al., 2005; Ahmadi et al., 2015). Bertocchi et al. (2011) found that atorvastatin (ATV) decreased TLR2 expression in a manner dependent on time and dosage, achieved by increasing CK2-mediated serine phosphorylation of Sp1 and suppressing Sp1’s ability to bind to DNA, ultimately leading to a decrease in the formation of atherosclerotic plaques.

Hypoxia-induced insulin resistance is promoted by the procoagulant molecule von Willebrand factor (vWF), which is produced more frequently during endothelial dysfunction and a prothrombotic state. Factors such as insulin resistance and elevated blood coagulation significantly contribute to atherosclerosis (Li et al., 2016; Asada et al., 2020). Singh et al. (2016) found that the HMGB1-TLR2-MYD88 pathway was upregulated in a mouse model of acute hypoxia (AH), with marked increases in Sp1 phosphorylation and vWF levels. Further studies demonstrated that silencing TLR2 inhibited Sp1 phosphorylation and prevented Sp1 from combining with the vWF promoter, thereby reducing vWF levels as well as alleviating AH-induced insulin resistance. This suggests that hypoxia-mediated enhancement of the TLR2-Sp1-vWF pathway plays a critical role in the etiology of atherosclerosis (Singh et al., 2016).

In conclusion, the mechanism underlying the progression of atherosclerotic plaque formation is intricately linked to the phosphorylation of Sp1. The upregulation of TF, P4Ha1, vWF and TLR2 has been shown to enhance Sp1 phosphorylation, thereby promoting atherosclerotic plaque development. Phosphorylated Sp1 can also initiate the transcription of the proapoptotic gene FasL in VSMCs, which compromises plaque stability and increases the risk of plaque rupture, thrombosis, and sudden death. Conversely, inhibition of Sp1 phosphorylation has been observed to reduce plaque formation. Therefore, targeting the phosphorylation of Sp1 represents a promising therapeutic strategy for mitigating atherosclerosis.

#### 3.1.2 Sp1 phosphorylation and lipid metabolism

There has been evidence that ATP-binding cassette transporter A1 (ABCA1) is positively correlated with cholesterol efflux from macrophages (Saleheen et al., 2015). PKCζ, part of the phospholipid-dependent serine/threonine kinase group, has the ability to attach to and add phosphate groups to the zinc finger region of Sp1 (Islam et al., 2018; Mehta et al., 2019). By enhancing Sp1 binding to the ABCA1 promoter, PI3K- and PKCζ-stimulated increases in Sp1 phosphorylation promote the expression of ABCA1. Interestingly, heat shock protein 27 (Hsp27) activates the PI3K/PKCζ pathway, promoting Sp1 phosphorylation in foam cells from THP-1 macrophages, thereby upregulating ABCA1 level and improving cholesterol excretion (Kuang et al., 2017; Chen et al., 2011). Additionally, R5-6, a ligand of the lipoprotein receptor and apolipoprotein E receptor-2 (apoER-2), facilitates ApoAI-mediated cholesterol export by upregulating ABCA1 expression. By inducing Sp1 phosphorylation, R5-6 enhances Sp1’s ability to bind to the ABCA1 promoter, increasing ABCA1 production as well as promoting cholesterol excretion (Okoro et al., 2015).

The plasma levels of low-density lipoprotein (LDL) can be elevated when LDL receptors (LDLR) are abnormally expressed, which is crucial for maintaining cholesterol homeostasis (Mineo, 2020). It is believed that Sp1 regulates the expression of the LDLR gene by binding to the promoter region of the gene (Jiang et al., 2022). Kakaferol has been found to promote Sp1 phosphorylation on Thr-453/739 sites via ERK protease, recruiting Sp1 to the LDLR promoter and enhancing LDLR expression, thus maintaining cholesterol homeostasis (Ochiai et al., 2016).

High blood triglycerides are a risk factor for atherosclerosis, characterized by elevated triglyceride-abundant lipoproteins in the plasma (Toth, 2021). Lipoprotein lipase (LPL) is essential for triglyceride hydrolysis and regulates circulating lipoprotein particles (Young et al., 2019). It was found that interferon-γ (IFN-γ) reduces Sp1’s ability to combine with the LPL promoter mediated through CK2, thereby reducing LPL gene transcription in macrophages. IFN-γ promotes CK2 activation and increases the interaction between Sp1 and CK2 catalytic subunits, leading to Sp1 phosphorylation and reduced binding to the LPL promoter (Hughes et al., 2002; Harris et al., 2008).

In summary, the maintenance of serum lipid homeostasis is crucial for the prevention of plaque formation. Elevated levels of LDL facilitate the transfer of cholesterol to peripheral cells, whereas phosphorylated Sp1 enhances LDL transcription. Additionally, the phosphorylation of Sp1 augments its binding affinity to the ABCA1 promoter, thereby upregulating ABCA1 expression and promoting cholesterol excretion. Conversely, INF-γ disrupts triglyceride metabolism by reducing Sp1 phosphorylation and its subsequent binding to the LPL promoter. Interestingly, the lipid-lowering agent atorvastatin has been observed to reduce atherosclerotic plaque formation by inhibiting Sp1 phosphorylation. This suggests that Sp1 phosphorylation is more closely associated with the development of atherosclerosis than with the maintenance of cholesterol stability.

#### 3.1.3 Sp1 phosphorylation and vascular endothelial function

Vascular Endothelial Growth Factor (VEGF), a powerful factor that promotes blood vessel growth, is implicated in multiple diseases and can be controlled by the activation of transcription factors like Sp1 (Bai and Chen, 2021; Pagès and Pouysségur, 2005). Dimethylarginine dimethylaminohydrolases (DDAHs), key enzymes degrading asymmetric dimethylarginine, include two isoforms: DDAH1 and DDAH2. DDAH2 induces VEGF expression by promoting Sp1 phosphorylation and enhancing Sp1’s transcriptional activity. Additionally, DDAH2 increases PKA activity, leading to Sp1 phosphorylation at threonine residues and higher nuclear Sp1 protein levels. These mechanisms synergize to increase Sp1 attachment to the VEGF gene promoter, thus promoting VEGF expression (Hasegawa et al., 2006).

Overexpression of heme oxygenase-1 (HO-1) also increases VEGF levels and angiogenesis in ischemic hearts (An et al., 2019). In a study by Lin et al. (2011), it was shown that HO-1/CO caused Sp1 phosphorylation on Thr-453/739 sites, subsequently inducing VEGF expression in cardiomyocytes. Nevertheless, the increase in VEGF production caused by HO-1/CO was notably reduced when a p38 kinase inhibitor was used, suggesting that HO-1/CO phosphorylates Sp1 via p38 kinase. Increased levels of Sp1 T453A or T739A variants decreased Sp1’s ability to bind to the VEGF promoter induced by HO-1/CO. The results imply that p38-mediated Sp1 phosphorylation on Thr-453/739 sites is necessary for production of VEGF induced by HO-1/CO in cardiomyocyte as well as angiogenesis (Lin et al., 2011). Furthermore, the pro-angiogenic hepatocyte growth factor (HGF/SF) causes Sp1 to be serine phosphorylated via PI3K, MEK1/2, and PKC-ζ kinase, enhancing Sp1’s transcriptional activity and upregulating VEGF/VPF production (Reisinger et al., 2003).

The eNOS/NO system is crucial for maintaining vascular integrity and regulating blood pressure (Dong et al., 2021; Garcia and Sessa, 2019). Cyanidin-3-glucoside (Cy3G), a natural dietary flavonoid, is believed to help prevent cardiovascular conditions by working as an antioxidant and vasodilator (Sivasinprasasn et al., 2016; Pantan et al., 2016). Xu et al. (2004) found that Cy3G increased eNOS/NO content in bovine arterial ECs in a time-dependent manner. Additionally, Cy3G enhanced Src and ERK1/2 phosphorylation over time. PP2, a Src kinase antagonist, and PD98059, a MEK antagonist inhibited eNOS production and ERK1/2 phosphorylation. Additionally, when Src was inactivated, Src-stimulated eNOS expression was reversed. These results indicate that Cy3G activates the Src-ERK1/2 kinase signaling cascade, thereby enhancing eNOS production. Further analysis revealed that Cy3G promoted Sp1 phosphorylation, increasing Sp1’s binding activity to eNOS, while PD98059 blocked Cy3G-stimulated Sp1 phosphorylation. These studies suggest that Cy3G elevates eNOS/NO levels in VECs through the Src-ERK1/2-Sp1 pathway, ameliorates endothelial dysfunction, regulates blood pressure, and protects against atherosclerosis.

In summary, the mechanism by which the eNOS/NO system ameliorates endothelial dysfunction involves the phosphorylation of Sp1. Phosphorylated Sp1 can upregulate eNOS expression, thereby maintaining vascular integrity and preventing atherosclerosis. Additionally, Sp1 phosphorylation enhances the expression of VEGF, which promotes angiogenesis. Angiogenesis within atherosclerotic plaques facilitates the transition from stable to unstable plaques and can precipitate plaque rupture. The HO-1/CO pathway induces VEGF production and subsequent angiogenesis, contingent upon the phosphorylation of Sp1 at Thr-453/739. Importantly, phosphorylation of Sp1 at these sites is also implicated in the regulation of cholesterol homeostasis. Consequently, therapeutic strategies targeting phosphorylated Sp1 in the treatment of atherosclerosis must carefully weigh the potential benefits against the associated risks, suggesting the necessity of identifying an optimal balance.

#### 3.1.4 Sp1 phosphorylation and VSMCs proliferation

The overexpression of the platelet-derived growth factor (PDGF-D) promoter leads to interstitial fibroblast proliferation, extensive collagen deposition, and arterial wall thickening, thereby promoting cardiac fibrosis and atherosclerosis (Folestad et al., 2018). In a ligation-stimulated rat carotid artery damage model, Tan et al. (2008) showed that upregulation of angiotensin-converting enzyme and PKC-ζ promoted Sp1 phosphorylation, enhancing Sp1’s binding activity to the PDGF-D promoter and subsequently increasing PDGF-D levels following acute vascular damage . Phosphorylation of Sp1 by PKC-ζ at Thr-668/681 and Ser-670 sites plays a vital role in Ang II-induced PDGF-D promoter activation in VSMCs. Further studies revealed that Ang II-stimulated phosphorylation of PKC-ζ and Sp1 is triggered through the Ang II type 1 receptor (AT1R). Ang II stimulates phosphorylation of PKC-ζ at Thr410, which enhances Sp1 phosphorylation and promotes its binding to the PDGF-D promoter, resulting in PDGF-D overexpression. Additionally, phosphorylation of Sp1 is observed in atherosclerotic plaques of VSMCs and dynamically expressed in injured rat carotid artery walls. These findings suggest that Ang II promotes PDGF-D expression through PKC-ζ-mediated Sp1 phosphorylation, contributing to vascular wall injury.

AT1R is involved Ang II-triggered contraction, proliferation, and migration of VSMCs (Kim and Kim, 2022; Dai et al., 2019). Activation of PPARγ could inhibit AT1R levels by reducing Sp1’s ability to attach to GC-box sequences of the AT1R promoter (Sugawara et al., 2001). Inhibiting Sp1 phosphorylation, p16 protein-dependent kinase inhibitors form a regulatory cycle that can be triggered by protein a/cyclin-dependent kinase (Wang et al., 2006).

Liver X receptors (LXRs) are nuclear hormone receptors involved in inflammation and lipid metabolism (Wang and Tontonoz, 2018). Imayama et al. (2008) showed that LXR agonist T0901317 increased p16, reduced Sp1 phosphorylation, and decreased AT1R expression, thereby inhibiting Sp1 function and VSMCs contraction, proliferation, and migration. Furthermore, fibroblast growth factor 2 (FGF-2) also regulates vascular lesions aside from PDGF. FGF-2 promotes VSMCs proliferation, with its levels decreasing as arterial plaque deteriorates, whereas PDGF-α and its receptor levels increase (Wang et al., 2022; Irvine et al., 2000). Milanini-Mongiat et al. (2002) found that originally protein kinase-activated phosphorylation of Sp1 at Thr-453/739 sites was mediated by p42/p44 MAPK. Bonello and Khachigian (2004) demonstrated that FGF-2 hinders the transcription of PDGFR-α by promoting Sp1 phosphorylation dependent on ERK1/2, consequently delaying plaque degradation. Sp1 attaches to an atypical region of PDGFR-α promoter, G10, to positively regulate PDGFR-α transcription. However, FGF-2-induced phosphorylation of Sp1 shifts this activation to inhibition. Chan et al. (2010) concluded that FGF-2 also attenuates arterial plaque by regulating TRAIL transcription. FGF-2 activates ERK1/2-mediated Sp1 phosphorylation at Thr453 and Thr739 sites, and phosphorylated Sp1 synergizes with NF-κB to bind the Oligo-1 recognition element of the TRAIL promoter, promoting TRAIL transcription and VSMCs proliferation.

Overall, these findings indicate that the effects of Sp1 phosphorylation on the contraction, proliferation, and migration of VSMCs during various stages of atherosclerosis are bidirectional. In the early phase of plaque formation, Sp1 phosphorylation elevates PDGF-D levels, which in turn drives VSMCs towards the inner membrane, resulting in diffuse intimal thickening. In the later stages, VSMCs proliferation contributes to plaque stabilization, with FGF-2 promoting VSMCs proliferation through the activation of ERK1/2-mediated phosphorylation of Sp1 at Thr-453/739. However, the expression of FGF2 diminishes as plaque progression worsens. Meanwhile, phosphorylated Sp1 can also initiate the transcription of the proapoptotic gene FasL in VSMCs, leading to a reduction in plaque stability. Phosphorylation at Thr-453/739 is implicated in various pathological processes, which may be an important breakthrough point.

#### 3.1.5 Sp1 phosphorylation and arrhythmia

Ventricular arrhythmias (VA) are more likely to occur after myocardial infarction (MI) due to a reduction in the Kir2.1 protein-mediated inward rectifying K+ current (IK1) (Vaidyanathan et al., 2018). Sp1, a crucial transcription factor for the Kir2.1-encoding KCNJ2 gene, is phosphorylated and promoted by CK2. Li et al. (2015) discovered that in rats with MI, CK2 expression was markedly elevated in H9c2 cardiomyocytes stimulated by hypoxia and along the margin of the infarcted myocardium. CK2-mediated Sp1 phosphorylation decreases its DNA-binding activity, suppressing KCNJ2 and IK1/Kir2.1 expression. However, the AT1R antagonist Valsartan can inhibit CK2 activation post-MI, eliminate CK2 phosphorylation of Sp1, increase Kir2.1 expression, and improve IK1 remodeling, suggesting a new treatment strategy for post-MI arrhythmia.

Numerous alkaloids have been shown to inhibit the cardiac hERG channel, predisposing patients to VA and sudden death (Litjens and Brunt, 2016; Coulson et al., 2017). Zhan et al. (2021) observed that the QT/QTc interval and incidence of ventricular fibrillation (VF) in guinea pig hearts were prolonged and elevated following a two-week treatment with Rutaecarpine (Rut), an extract of Evodia officinarum. Subsequent research revealed that Rut lowered PI3K/Akt pathway-mediated threonine (Thr)/tyrosine (Tyr) phosphorylation of Sp1, reducing hERG protein expression and inhibiting the hERG current (IhERG), thereby increasing the risk of VA and sudden death (Zhan et al., 2021).

**FIGURE 2:**
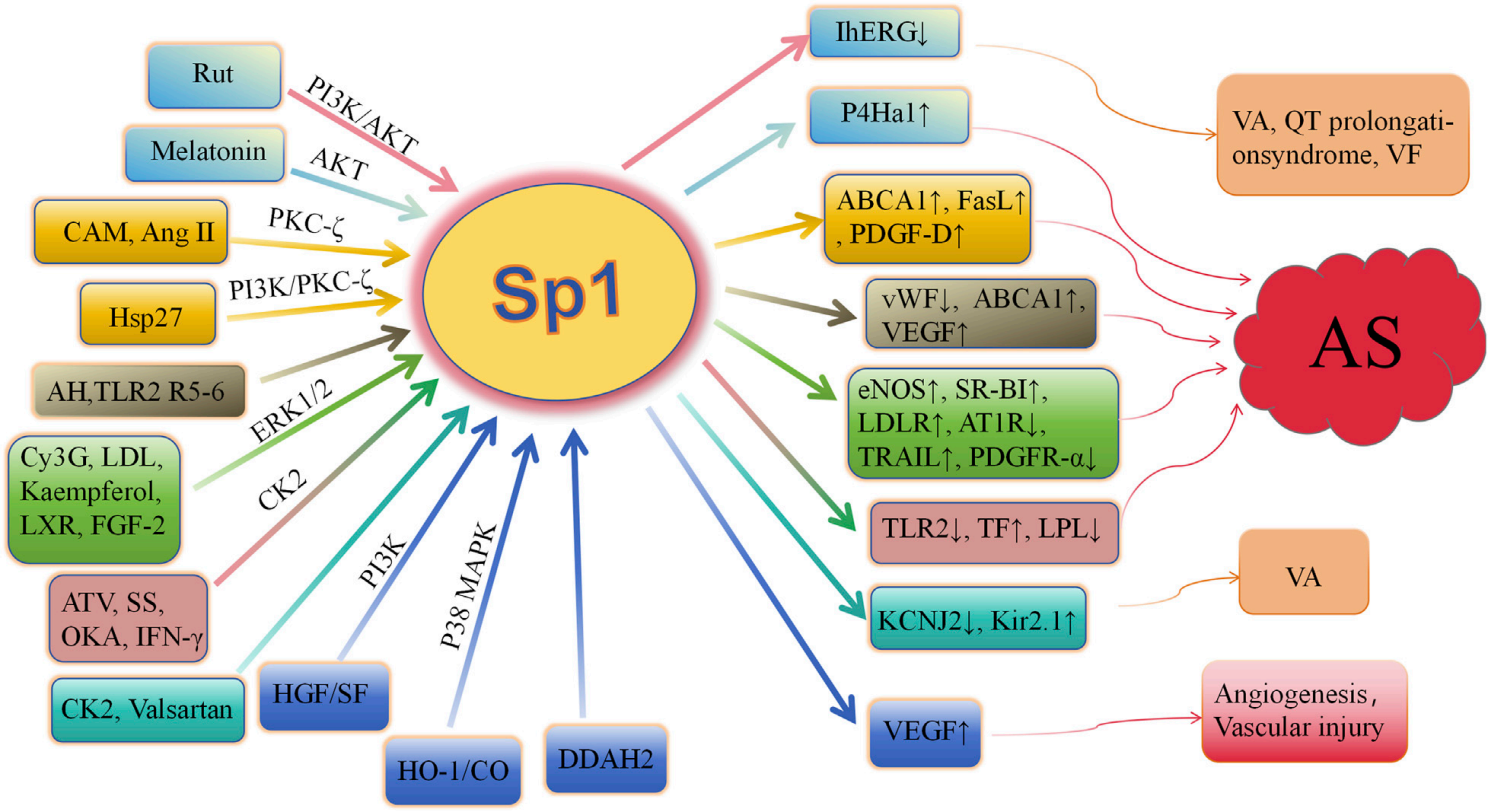
Role of Sp1 phosphorylation in cardiovascular diseases AS, arteriosclerosis; Rut, rutaecarpine; Hsp27, heat shock protein 27; AKT, protein kinase B; PKC ζ, protein kinase C ζ; CAM, camptothecin; Ang II, angiotensin II; AH, acute hypoxia; R5-6, Reelin 5-6; Cy3G, cyanidin-3-glucoside; LDL, low-density lipoprotein; LXR, liver X receptor; FGF-2, fibroblast growth factor-2; HO-1, heme oxygenase-1; ATV, atorvastatin; CK2, casein kinase 2; SS, shear stress; IFN-γ, interferon-γ; IhERG, hERG current; ERK, extracellular signal-regulated kinase; p38 MAPK, p38 mitogen-activated protein kinase; P4Ha1, prolyl-4-hydroxylase α1; ABCA1, ATP-binding cassette transporter A1; FasL, fas ligand; PDGF-D, platelet-derived growth factor-D; vWF, von willebrand factor; SR-BI, scavenger receptor class B type I; LDLR, low-density lipoprotein receptor; TRAIL, tumor necrosis factor–related apoptosis-inducing ligand; PDGFR-α, platelet-derived growth factor-α; TF, tissue factor; VEGF, vascular endothelial growth factor; TLR2, toll-like receptor-2; LPL, lipoprotein lipase; VA, ventricular arrhythmias; VF, ventricular fibrillation; HGF, hepatocyte growth factor; OKA, okadaic acid; DDAH, dimethylarginie dimethylaminohydrolase.

Consequently, a growing number of kinases, such as Akt, PKCζ, ERK1/2, CK2, and PI3K, have been implicated in the induction of Sp1 phosphorylation. This phosphorylation of Sp1 facilitates the expression of specific genes, including LDLR, TF, TLR2, ABCA1, P4Ha1, and vWF. A significant correlation exists between Sp1 phosphorylation and the development of atherosclerosis (Figure 2). Sp1 phosphorylation plays a crucial role in mitigating atherosclerotic plaque formation, contributing plaque stability, maintaining cholesterol homeostasis (Thr453/739), and regulating to endothelial dysfunction. Comprehending the regulatory mechanisms of phosphorylation is vital for understanding protein function and disease processes. More research may identify additional phosphorylation targets and their roles in biological processes, providing new avenues for drug discovery and advancing the management and prevention of cardiovascular disorders. Clarifying how phosphorylation affects this crucial transcription factor’s function in both health and disease is essential.

### 3.2 Acetylation

The acetylation process adds acetyl groups to target proteins at their lysines or N-termini. This highly conserved and reversible PTM is essential for maintaining stability of proteins. Acetylation and deacetylation, regulated by lysine deacetylases and lysine acetyltransferases, alter protein function, thereby influencing gene expression and the regulation of autophagy initiation and selective autophagy by modulating the acetylation levels of key autophagy proteins (Shvedunova and Akhtar, 2022; Yiew et al., 2015). Population aging leads to an increasing incidence of vascular calcification (VC), in which Sp1 plays a significant role (Zhang et al., 2018; Li Y. et al., 2024).

**FIGURE 3:**
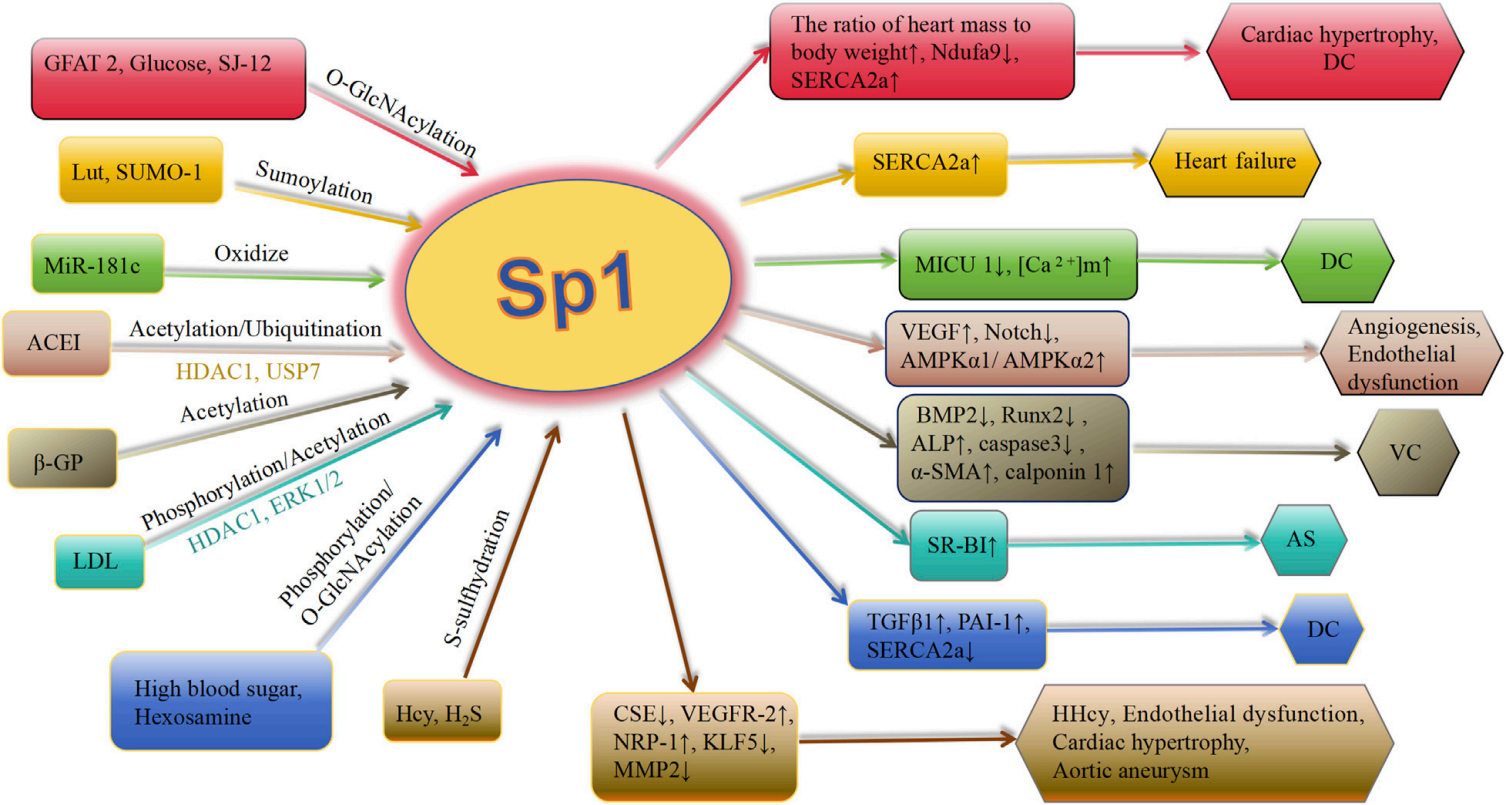
Schematic association of PTMs (in addition to phosphorylation) of Sp1 with cardiovascular diseases. Lut, luteolin; SUMO-1, small ubiquitin-related modifier-1; ACEI, angiotensin-converting enzyme inhibitor; Ang II, angiotensin II; ET-1, endothelin-1; VC, vascular calcification; HDAC 1, histone deacetylases 1; ACEI, angiotensin-converting enzyme inhibitor; CSE, cystathionine γ-lyase; SERCA2a, asarcoendoplasmic reticulum Ca(2+)-ATPase 2a; TGFβ1, transforming growth factor-β1; PAI-1, plasminogen activator inhibitor-1; GFAT 2, glutamine-fructose amidotransferase 2; OGA, O-GlcNAcase; MICU 1, mitochondrial calcium uptake 1 protein; [Ca 2^+^]m, mitochondrial calcium level; AMPK, AMP-activated protein kinase; BMP 2, bone morphogenetic protein 2; VEGFR-2, vascular endothelial growth factor receptor-2; NRP-1, neuropilin-1; KLF5, krüppel-like factor 5; Hcy, homocysteine; MMP 2, metalloproteinases 2; DC, diabeticcardiomyopathy; AS, arteriosclerosis; HHcy, hyperhomocystinemia; ALP, alkaline phosphatase; Runx2, runt-related transcription factor 2; α-SMA, α-smooth muscle actin; β-GP, β-glycerophosphate; LDL, low-density lipoprotein.

Deacetylation of Sp1 prevents it from attaching to the promoters of genes further downstream, leading to a decrease in their expression. Bone morphogenetic proteins (BMPs), involved in arterial remodeling, show increased expression in calcified arterial lesions (Lu et al., 2024; Hao et al., 2023). Apoptosis of VSMCs is a key event in VC. Zhang et al. (2021) discovered an increase in the acetylation level of Sp1 at Lys704 in a β-glycerophosphoric acid-stimulated VSMC calcification model. Moreover, interfering with Sp1 acetylation using the Sp1-K704A mutant plasmid showed that deacetylating Sp1 down-regulated osteogenic markers Runx2 and BMP2, reduced alkaline phosphatase (ALP) activity and calcium content, inhibited caspase-3 activity, and prevented VSMCs apoptosis. Additionally, the expression of α-SMA and calponin 1 was upregulated. Decreased attachment of deacetylated Sp1 to the BMP2 gene promoter leads to a decrease in BMP2 production, resulting in decreased cell death, change in cell characteristics, and calcium buildup in calcified VSMCs (Zhang et al., 2021) (Figure 3). In general, during atherosclerosis, VSMCs can differentiate into multiple phenotypes, including calcified cells. In the calcification model of VSMCs, the level of acetylated Sp1 is elevated, whereas interference with Sp1 acetylation inhibits both phenotypic transformation and apoptosis of VSMCs, thereby modulating calcification. This suggests that Sp1 acetylation could be a potential therapeutic target for VC.

### 3.3 S-sulfhydration

S-sulfhydration converts cysteine-SH groups to -SSH via REDOX by H_2_S or persulfides (Song et al., 2023; Li Z. et al., 2024). Sp1, containing 11 cysteine residues, undergoes S-sulfhydration, which is crucial to the regulation of gene transcription in the vascular system (Zhu et al., 2022). This novel PTM significantly impacts cardiovascular diseases by influencing cell apoptosis, proliferation, vasodilation, anti-inflammatory response, and oxidative stress (Li Z. et al., 2024). Varying effects on Sp1 activity arise from different cysteine residues undergoing S-sulfhydration, indicating distinct biological roles (Xie et al., 2016; Luo et al., 2021).

Homocysteine (Hcy) undergoes trans-sulfuration via H2S. Cystathionine gamma-lyase (CSE), the primary H2S-producing enzyme in the cardiovascular system, is crucial for Hcy metabolism (Yang and He, 2019; Fan et al., 2019). Luo et al. (2021) established a hyperhomocysteinemia (HHcy) model using a high-methionine diet, discovering that Hcy accumulation impaired CSE activity, increased CSE nitration, and reduced H2S production. This deficiency in H2S diminished Sp1 S-sulfhydration and its binding to the CSE promoter, downregulating CSE and further decreasing H_2_S, thus disrupting the Hcy trans-sulfuration pathway and raising serum Hcy levels. The H_2_S donor GYY4137 lowered homocysteine levels, increased Sp1 S-sulfhydration levels, and restored Sp1-CSE-H_2_S pathway function. These insights reveal a novel mechanism for treating severe HHcy.

Deficiency of human cystathionine β-synthase (CBS) leads to VECs dysfunction (Rao et al., 2020; Lv et al., 2020). Saha et al. (2016) found that H_2_S produced by CBS in VECs stabilizes Sp1. H_2_S modifies the Cys68 and Cys755 sites of Sp1 through S-sulfhydration, enhancing Sp1 binding to VEGFR-2, upregulating VEGFR-2 and NRP-1 levels, and thereby improving VEGF-induced endothelial responses to treat CBS deficiency-induced dysfunction. This highlights the significance of CBS-facilitated S-sulfhydration in preserving vascular well-being.

Additionally, S-sulfhydration of Sp1 prevents cardiac hypertrophy by regulating angiotensin. Krüppel-like factor 5 (KLF5) is essential in the development of hypertrophic cardiomyopathy (Shindo et al., 2002). Meng et al. (2016) reported that CSE expression is downregulated while KLF5 expression is elevated in hypertrophic myocardial tissue and Ang II-treated cardiomyocytes in hypertensive rats. Administration of GYY4137, an H_2_S donor, inhibited cardiac hypertrophy and downregulated KLF5 protein levels. H_2_S S-sulfhydrated Sp1 at Cys664 site, inhibiting Sp1 binding to the KLF5 promoter, thereby downregulating KLF5 transcription and preventing cardiac hypertrophy (Meng et al., 2016).

During an aortic aneurysm, matrix metalloproteinases (MMPs) degrade elastin, causing the aortic wall to dilate permanently (Wang and Khalil, 2018). Zhu et al. (2022) discovered that older mice lacking CSE show significant enlargement of the aorta and deterioration of elasticity, making them more vulnerable to aortic damage caused by Ang II. Exogenous H_2_S donor NaHS protected mice from Ang II-stimulated elastin fragmentation and aortic dilation. H_2_S modifies Sp1 via S-sulfhydration, reducing Sp1 binding to the MMP2 promoter, thereby decreasing MMP2 expression, maintaining elastin levels, and reducing aortic aneurysm pathology (Zhu et al., 2022). In conclusion, S-sulfhydration of Sp1 is crucial for H2S’s cardiovascular safeguarding effects.

CSE can metabolize Hcy to H_2_S; however, excessive accumulation of Hcy impairs CSE activity, leading to a reduction in H_2_S production. This deficiency in H_2_S results in decreased S-sulfhydration of Sp1, which subsequently downregulates CSE expression and exacerbates the reduction in H_2_S production and Hcy accumulation, thereby creating a vicious cycle. H_2_S donors can disrupt this cycle by restoring the S-sulfhydration of Sp1 and reversing serum Hcy levels through increased CSE transcription. Furthermore, H_2_S can upregulate VEGFR-2 via the S-sulfhydration of Sp1, promoting the proliferation and migration of VECs, and thereby ameliorating endothelial dysfunction induced by CBS deficiency. S-sulfhydration not only enhances Sp1 binding to the promoter regions of CSE and VEGFR-2 but also diminishes Sp1 binding to the promoters of KLF5 and MMP2. Consequently, the expression of KLF5 and MMP2 is downregulated, leading to the suppression of cardiac hypertrophy and the attenuation of pathological changes in aortic aneurysms, respectively. These findings suggest that S-sulfhydrated Sp1 exhibits binding selectivity, which could be exploited for the development of targeted therapeutic agents.

### 3.4 O-GlcNAcylation

O-GlcNAcylation is a nutritionally sensitive and conserved PTM capable of influencing gene transcription and cell signaling pathways (Yue et al., 2023). O-linked N-acetylglucosamine transferase (OGT) is required for this process, as it utilizes UDP-GlcNAc, a product of hexosamine biosynthesis, to modify serine and threonine sites, similarly to protein phosphorylation. However, O-GlcNAcylation can be reversed by O-GlcNAcase (OGA), a single glycoside hydrolase (Belke, 2011; Chen et al., 2018). Elevated protein O-GlcNAcylation levels in myocardial tissue are associated with diabetes, ischemic cardiomyopathy, and pathological hypertrophy, whereas exercise-induced normal hypertrophy is linked to decreased protein O-GlcNAcylation (Belke, 2011; Young et al., 2007; Han and Kudlow, 1997).

Research has shown that Sp1 deficiency induces hypertrophic cardiomyopathy (HCM), while Sp1 overexpression holds therapeutic potential for HCM by ameliorating pathological characteristics in HCM mouse models, such as cardiomyocyte hypertrophy and muscle fiber disorders (Zhang et al., 2024). Research has demonstrated that swimming exercise can lead to physiological enlargement of the heart in mice, resulting in enhanced heart muscle function (Kim et al., 2008). Belke (2011) found that swimming training significantly reduced Sp1 O-GlcNAcylation levels in mice compared to sedentary mice. This reduction is due to lower glutamine-fructose-6-phosphate aminotransferase 2 mRNA levels and higher OGA levels, resulting in weakened Sp1 O-GlcNAcylation. Consequently, this reduction increases the heart weight-to-body weight ratio, improves systolic performance, promotes cardiac remodeling, and induces physiological cardiac hypertrophy (Belke, 2011).

Impaired cardiac contractility is the main pathological feature of diabetic cardiomyopathy (Dillmann, 2019). Patients with poorly managed diabetes exhibit elevated fatty acid consumption by the heart and reduced glucose consumption. GLUT4 is primarily responsible for facilitating myocardial basal glucose uptake during contraction. However, GLUT4 inhibition in diabetic cardiomyopathy suggests that maintaining or increasing cardiac GLUT4 levels may help sustain myocardial glucose utilization (Wende et al., 2020).

The increased activity of Sp1 after O-GlcNAcylation contributes significantly to the cardiovascular impairment of hyperglycemia-stimulated glucotoxicity (Paueksakon et al., 2002). By constructing a mouse model with overexpressed GLUT4, Wende et al. (2020) demonstrated that increased myocardial glucose uptake promotes extensive O-GlcNAcylation of Sp1 in diabetes. This modification suppresses the production of Ndufa9, a subunit of mitochondrial complex I, in a manner dependent on glucose and O-GlcNAc, leading to higher glucose levels and increased susceptibility of the heart to glucotoxicity. This exacerbates mitochondrial dysfunction and diabetic cardiomyopathy (Wende et al., 2020).

Furthermore, the clearance of cytoplasmic calcium during cardiac contraction is contingent upon Sarcoplasmic/Endoplasmic Reticulum Calcium ATPase 2a (SERCA2a), whose gene expression in cardiomyocytes is regulated by the transcription factors Sp1 and Sp3. These factors are crucial for SERCA2a expression during both cardiac development and disease (Brady et al., 2003). Lou et al. (2024) found that a novel curcumin analogue SJ-12 effectively suppresses myocardial hypertrophy and fibrosis in streptozotocin-induced diabetic cardiomyopathy mice. Mechanistically, SJ-12 lowered the Sp1 O-GlcNAcylation through blocking OGT to stabilize SERCA2a, mitigating myocardial fibrosis and hypertrophy.

In conclusion, the modulation of Sp1 O-GlcNAcylation levels in cardiomyocytes significantly influences cardiac remodeling. Specifically, reduced levels of Sp1 O-GlcNAcylation are associated with physiological cardiac hypertrophy, which typically regresses with decreased exercise intensity. Conversely, elevated Sp1 O-GlcNAcylation levels exacerbate mitochondrial dysfunction and contribute to pathological cardiac hypertrophy and myocardial fibrosis in patients with diabetic cardiomyopathy. Inhibition of Sp1 O-GlcNAcylation effectively mitigates maladaptive cardiac remodeling. The protective effect of exercise on diabetic cardiomyopathy may be attributed to the reduction of Sp1 O-GlcNAcylation levels.

### 3.5 SUMOylation

SUMO, a protein modifier that is similar to ubiquitin, is present in many eukaryotic organisms and was initially identified in 1996. SUMOylation, a reversible PTM, plays a crucial role in regulating DNA damage repair, immune response, cancer development, cell cycle advancement, and programmed cell death. By attaching SUMO to specific lysine residues in substrate proteins through the actions of E1 activating enzyme, E2 conjugating enzyme, and E3 ligase, SUMOylation influences the intracellular distribution, conformation, stability, and other modifications of the substrate protein (Han et al., 2018; Zhao et al., 2021; Chen et al., 2020; Liu et al., 2021).

When calcium reuptake occurs during excitation-contraction coupling, SERCA2a plays an important role. *In vitro* and *in vivo*, reduced SERCA2a activity leads to impaired SER Ca^2+^ uptake, leading to significant contractile abnormalities. Studies have demonstrated that enhancing SERCA2a levels can enhance heart failure (HF) and cardiac function (Zhihao et al., 2020; Gotthardt and Lehnart, 2024; Arici et al., 2024). Tilemann et al. (2013) demonstrated that SUMO-1 overexpression in cardiomyocytes increases Sp1 SUMOylation and upregulates SERCA2a transcript levels in MI-induced failing hearts, thereby ameliorating HF and cardiac function. Therefore, targeting Sp1 SUMOylation could be an effective approach for treating HF. Luteolin (Lut), a type of flavonoid, is crucial in the prevention of cardiovascular diseases (Huang et al., 2023; Wang et al., 2023). Hu et al. (2017) found that in Lut-treated cardiomyocytes, the PI3K/Akt pathway was activated and SUMO-1 expression significantly increased. Lut also enhanced the SUMOylation of Sp1, which stimulated SERCA2a gene expression, increasing intracellular Ca^2+^ levels. However, the Akt inhibitor reversed Lut’s effects, suggesting that Lut promotes SUMO-1 expression and increases Sp1 SUMOylation by activating the PI3K/Akt pathway. This activation enhances Sp1 binding to the SERCA2a promoter, promoting its transcription and alleviating HF dysfunction.

The current study indicates that SUMOylation of Sp1 is predominantly associated with SERCA2a. Sp1 SUMOylation enhances its binding to the SERCA2a promoter, leading to an upregulation of SERCA2a, which subsequently increases intracellular Ca^2+^ uptake. This mechanism not only ameliorates MI-induced HF but also mitigates cardiac diastolic dysfunction resulting from I/R injury, presenting a potential therapeutic target for HF.

### 3.6 Oxidative

Reactive oxygen species (ROS), including H_2_O_2_, stimulate oxidation of important redox-sensitive cysteine residues on specific proteins (Lennicke and Cochemé, 2021). Such oxidative PTMs regulate the biological activities, cellular localization, and interactions of various enzymes and transcription factors. Dysregulated redox homeostasis can lead to various diseases. ROS have the ability to attach to the cysteine residues located in the zinc finger domain of Sp1, which then undergo PTMs that impact the activity of Sp1 (Hopkins and Neumann, 2019).

Mitochondrial calcium uptake 1 protein (MICU1) is a regulatory protein in the mitochondrial calcium uniporter complex, which regulates mitochondrial calcium [(Ca2+)m] levels in response to stress from obesity and type 2 diabetes (Shi et al., 2023; Kaye et al., 2024). Sp1 directly binds to the MICU1 promoter region, regulating its expression (Banavath et al., 2019; Ji et al., 2017). A high-fat diet (HFD) significantly upregulated miR-181c and ROS levels in the mouse heart, leading to cardiac hypertrophy, according to Roman et al. (2020). However, miR-181c knockout mice showed attenuated increases in these levels. HFD-fed miR-181c knockout mice had higher MICU1 levels than HFD-fed wild-type mice, reducing the elevation in [Ca^2+^]m. Heightened ROS generation in cardiomyocytes caused by miR-181c overexpression oxidized and decreased Sp1 levels, reducing MICU1 transcription. These findings suggest that miR-181c-induced obesity is mediated by ROS-induced Sp1 oxidation, which reduces MICU1 expression. Protecting against miR-181c oxidation by inhibiting Sp1 offers potential therapeutic targets for obesity or type 2 diabetes cardiomyopathy.

As a novel PTM associated with cardiomyopathy, oxidation warrants significant attention. The normal uptake of mitochondrial Ca^2+^ facilitates increased ATP synthesis, inhibits autophagy, and buffers cytosolic Ca^2+^. Elevated ROS levels due to a HFD enhance Sp1 oxidation, thereby inhibiting MICU1 transcription and reducing Ca^2+^ uptake.

### 3.7 Sp1 crosstalk between different PTMs

Waby et al. (2008) demonstrated that different PTMs of Sp1, such as phosphorylation, acetylation, and SUMOylation, collaborate to create minor alterations in transcriptional activation. For instance, Sun et al. (2022) found that acetylation not only upregulates Sp1 protein levels by boosting its stability but also improves Sp1 stability by competitively blocking the ubiquitin-induced proteasomal degradation pathway. This interplay is reflected in the pathogenesis of cardiovascular disease. The pathomechanism of cardiovascular disease is complex and multifaceted, with common coordination between acetylation, ubiquitination, and phosphorylation.

#### 3.7.1 Acetylation and ubiquitination

Controlling blood pressure and vascular tone is a critical function of the vascular endothelium. Endothelial dysfunction is a significant cause of hypertension and a risk factor for developing microvascular and macrovascular complications. ACE inhibitors (ACEI) are commonly used to treat cardiovascular issues and have shown beneficial effects on promoting *in vivo* angiogenesis (Yasumatsu et al., 2004). Sp1 is regulated by ACEI via the translocation of the deubiquitinating enzyme USP7 and the acetylase HDAC1, which move to the nucleus and deacetylate Sp1. Lu et al. (2023b) found that endothelial Sp1 signaling is critical in the pathophysiological angiogenesis associated with ACEI treatment. Specifically, ACEI inhibits the acetylation of Sp1 at lysine 703 through HDAC1, promotes the interaction between Sp1 and USP7, inhibits Sp1 ubiquitination and degradation, and enhances its expression. ACEI promotes Sp1/Sp3 through crosstalk with VEGF and Notch signaling pathways to facilitate angiogenesis, which simultaneously increases VEGF signaling and inhibits Notch activation.

Similar mechanisms of acetylation and ubiquitination are associated with the pathogenesis of hypertension and endothelial abnormality. Lu et al. (2023a) also discovered that ACEI’s ability to lower blood pressure and improve endothelial function is related to the activation of endothelial Sp1. Consistent with previous research, ACEI enhanced Sp1 expression by USP7-mediated deubiquitination and HDAC1-mediated deacetylation. This promoted Sp1 binding to the promoters of AMPKα1 and AMPKα2, increased their transcription levels, and improved endothelial function via AMPKα. According to these results, Sp1 is a potentially novel therapeutic target for ACEIs in preventing cardiovascular disease.

#### 3.7.2 Phosphorylation and acetylation

Elevated plasma concentrations of LDL are significant risk factors for atherosclerosis, while higher levels HDL cholesterol concentrations are strongly inversely associated with the risk of coronary heart disease (Carr et al., 2019; Qin, 2020). Scavenger receptor class B type I (SR-BI), a vital HDL receptor, is essential for the liver’s absorption of HDL cholesterol and is crucial for facilitating the transport of cholesterol in reverse. The absence of SR-BI expression can lead to extensive atherosclerosis in mice (Linton et al., 2017). Yang et al. (2016) found that elevated LDL levels stimulated ERK1/2-kinase-mediated phosphorylation of Sp1, leading to the binding of histone acetyltransferase (HAT) p300 to the Sp1 transcription complex in the SR-BI promoter area and resulting in the detachment of deacetylase HDAC1 from the Sp1 transcription complex. This process increases histone acetylation, thereby inducing SR-BI gene transcription, upregulating SR-BI expression, and alleviating atherosclerosis. Furthermore, a novel phosphorylation site, Ser702, was discovered on Sp1. During the activation of SR-BI induced by LDL, there is also slight acetylation at the Lys703 locus within the third zinc finger region of Sp1.

#### 3.7.3 Phosphorylation and O-GlcNAcylation

High glucose or glucosamine can affect cardiovascular function by regulating Sp1 to increase the expression of plasminogen activator inhibitor-1 (PAI-1) and TGFβ, both of which are involved in atherosclerosis (Paueksakon et al., 2002; Issad and Kuo, 2008).

Du et al. (2000) demonstrated that elevated blood sugar levels lead to excessive production of superoxide in mitochondria, which in turn boosts the synthesis of hexosamine and O-GlcNAcylation of Sp1, ultimately triggering the activation of genes related to the development of diabetic complications. The specific mechanism involves high blood sugar inducing excessive superoxide production, redirecting fructose-6-phosphate away from glycolysis towards the hexosamine biosynthetic pathway, increasing hexosamine activity, and raising Sp1 O-GlcNAcylation. This reduces phosphorylation levels of Sp1 at Ser/Thr, further increasing TGFβ1 and PAI-1 transcription, leading to decreased cardiac systolic function and accelerating diabetic cardiomyopathy progression.

Clark et al. (2003) showed that the significance of the hexosamine pathway and O-GlcNAcylation in the deterioration of cardiomyocyte function and the progression of diabetic cardiomyopathy. Additionally, it was discovered that the SERCA promoter harbors numerous Sp1 binding sites. Increased Sp1 O-GlcNAcylation downregulates SERCA2a activity and expression, impairing sarcoendoplasmic reticulum (SR) function, leading to abnormal cardiac Ca^2+^ processing and decreased cardiac systolic function.

Further investigations into the dynamic interactions among various PTMs of Sp1 are essential for elucidating the molecular mechanisms underlying disease processes. Notably, acetylation and ubiquitination have been implicated in the pathogenesis of hypertension and endothelial abnormalities. Given that silencing or suppression of the Sp1 gene can result in significant endothelial dysfunction and hypertension, it is posited that endothelial aging and apoptosis may be mitigated by augmenting Sp1 expression through USP7-mediated deubiquitination and HDAC1-mediated deacetylation.

### 3.8 DNA methylation

Interestingly, in addition to the aforementioned protein PTMs concerning cardiovascular disease, Sp1 has a modification mode, namely DNA methylation. It is a stably inherited epigenetic modification that occurs in the CpG island of DNA (CG sequence dense region) (Moore et al., 2013).

Research studies have demonstrated a strong link between a negative fetal environment and a higher likelihood of developing ischemic heart disease in adulthood. Hypoxia during pregnancy is a frequent cause of stress for the fetus, resulting in reduced levels of cardiac PKCε and making the adult offspring heart more vulnerable to ischemia-reperfusion damage (Li et al., 2003). Patterson et al. (2010) showed that hypoxia led to higher levels of Sp1 methylation in fetal cardiomyocytes, and further found that this methylation prevented Sp1 from attaching to the PKCε promoter within chromatin, ultimately resulting in decreased levels of PKCε protein. Meyer et al. (2009) also found that cocaine-stimulated increments in Sp1 methylation similarly led to a reduced Sp1’s ability to bind to the PKCε promoter, ultimately resulting in a suppression in PKCε levels in the fetal heart.

## 4 Conclusion and perspective

Recent research has emphasized the crucial function of Sp1 in regulating the advancement of cardiovascular diseases. Sp1 is a significant transcription factor that controls numerous genes, including both positive regulatory genes and housekeeping genes. This review focuses on the PTMs of Sp1, such as phosphorylation, S-sulfhydrylation, SUMOylation, glycosylation, and acetylation, and their roles in regulating Sp1 stability and target gene expression in cardiovascular disease progression.

For example, PTMs of Sp1 are crucial in conditions like atherosclerosis, hypertension, heart disease, high homocysteine levels, and aortic aneurysms, as well as in inflammation, angiogenesis, plaque stability, endothelial dysfunction, lipid metabolism, and the pathological physiology of VSMCs, including apoptosis. Additionally, this review includes newly discovered modification patterns, modification sites, and their effects on the expression of key proteins (Table 1). These studies provide new targets and a conceptual foundation for addressing cardiovascular conditions through Sp1 PTMs.

**TABLE 1:** Post-translational modifications of Sp1 in cardiovascular diseases.

Modification	Materials	Kinases	Sites	Function	Diseases	References
Phosphorylation	SS, OKA	—	—	TF↑	AS	Lin et al. (1997)
	Melatonin	Akt	Ser42, Thr679, Ser698	P4Ha1↑		Li et al. (2019)
	CAM	PKC-ζ	—	FasL↑, apoptotic process↑		Bertocchi et al. (2011)
	ATV	CK2	—	TLR2↓		Bertocchi et al. (2011)
	AH, TLR2	—	—	vWF↓		Singh et al. (2016)
	Hsp27	PI3K/PKCζ PKCζ	—	ABCA1↑ ABCA1↑		Kuang et al. (2017) Chen et al. (2011)
	R5-6	—	—	ABCA1↑		Okoro et al. (2015)
	Kaempferol	ERK1/2	Thr-453, Thr-739	LDLR↑		Ochiai et al. (2016)
	FGF-2	ERK1/2	—	PDGFR-α↓		Bonello and Khachigian (2004)
	FGF-2	ERK1/2	Thr-453, Thr-739	TRAIL↑		Chan et al. (2010)
	IFN-γ IFN-γ	CK2 CK2	—	LPL↓ LPL↓		Hughes et al. (2002) Harris et al. (2008)
	Rut	PI3K/AKT	—	IhERG↓	VA, VF, QT prolongati-onsyndrome	Zhan et al. (2021)
	CK2, Valsartan	—	—	KCNJ2↓, Kir2.1↑	VA	Li et al. (2015)
	HGF/SF	PI3K	—	VEGF↑	Angiogenesis Angiogenesis	Reisinger et al. (2003)
	HO-1/CO	P38 MAPK	Thr-453, Thr-739			Lin et al. (2011)
	Ang II	PKC-ζ	Thr668, Ser670, Thr681	PDGF-D↑	Vascular injury	Tan et al. (2008)
	LXR	—	—	AT1R↓		Imayama et al. (2008)
	DDAH2	—	—	VEGF↑		Hasegawa et al. (2006)
	Cy3G	ERK1/2	—	eNOS↑	Endothelial dysfunction	Xu et al. (2004)
O-GlcNAcylation	GFAT 2	OGA	—	The ratio of heart mass to body weight↑	Cardiac hypertrophy	Belke (2011)
	Glucose	—	—	Ndufa9↓	DC	Wende et al. (2020)
	SJ-12	OGT	—	SERCA2a↑	DC	Lou et al. (2024)
Acetylation	β-GP	—	Lys704	BMP2↓, Runx2↓, ALP↑, caspase3↓, α-SMA↑, calponin 1↑	VC	Zhang et al. (2021)
S-sulfhydration	Hcy	—	—	CSE↓	HHcy	Luo et al. (2021)
	H_2_S	—	Cys68, Cys755	VEGFR-2↑, NRP-1↑	Endothelial dysfunction	Saha et al. (2016)
		—	Cys664	KLF5↓	Cardiac hypertrophy	Meng et al. (2016)
		—	—	MMP2↓	Aortic aneurysm	Zhu et al. (2022)
SUMOylation	—	SUMO-1	—	SERCA2a↑	Heart failure	Tilemann et al. (2013)
	Lut	PI3K/Akt	—			Hu et al. (2017)
Oxidize	MiR-181c	—	—	MICU 1↓, [Ca^2+^]m↑	DC	Roman et al. (2020)
Acetylation and ubiquitination	ACEI	HDAC1, USP7	Lys703	VEGF↑, notch↓	Angiogenesis	Lu et al. (2023b)
				AMPKα1/AMPKα2↑	Endothelial dysfunction	Lu et al. (2023a)
Phosphorylation and acetylation	LDL	HDAC1, ERK1/2	Ser702	SR-BI↑	AS	Yang et al. (2016)
Phosphorylation and O-GlcNAcylation	High blood sugar	—	—	TGFβ1↑, PAI-1↑, SERCA2a↓	DC	Du et al. (2000)
	Hexosamine	—	—	SERCA2a↓		Clark et al. (2003)

SS, shear stress; OKA, okadaic acid; LPL, lipoprotein lipase; VA, ventricular arrhythmias; VF, ventricular fibrillation; DC, diabetic cardiomyopathy; VC, vascular calcification.

Sp1, as a crucial transcription factor, regulates genes involved in various cardiovascular diseases. In atherosclerosis, Sp1 PTMs regulate genes such as ABCA1, VEGF, SR-BI, LDLR, TF, TLR2, P4Ha1, and AT1R. Sp1 phosphorylation, the most extensively studied modification, is mediated by several kinases. For instance, the enhancement of Sp1 phosphorylation and NFκB interaction through the FGF-2/FGF receptor pathway by ERK kinase-mediated FGF-2, leading to a increment in TRAIL transcription, mRNA, and protein expression, ultimately contributing to VSMCs proliferation and neointima formation following arterial damage (Chan et al., 2010). FGF-2 stimulation of ERK1/2 kinase-mediated Sp1 phosphorylation inhibits PDGFR-α transcription, slowing atherosclerotic plaque deterioration (Bonello and Khachigian, 2004). ERK kinase also promotes cyanidin-3-glucoside (Cy3G)-induced Sp1 phosphorylation, resulting in increased eNOS expression and NO production through the Src-ERK1/2-Sp1 pathway in VECs, which improves endothelial dysfunction, regulates blood pressure, and prevents atherosclerosis. Cy3G works with LDL to upregulate SR-BI expression, promoting reverse cholesterol transport, essential for preventing atherosclerosis and coronary artery disease. Sp1 and kaempferol synergistically recruit to the LDLR promoter to enhance LDLR expression and maintain cholesterol homeostasis (Ochiai et al., 2016). The LXR agonist T0901317 upregulates p16, reducing Sp1 phosphorylation, downregulating AT1R expression, and inhibiting Sp1, thereby reducing VSMC contraction, proliferation, and migration (Imayama et al., 2008). IFN-γ disrupts lipid metabolism by reducing Sp1 binding to the LPL promoter, promoting high blood triglycerides (Hughes et al., 2002). The AT1 receptor antagonist valsartan decreases CK2 activation, downregulates Sp1 phosphorylation, and increases Kir2.1 expression post-myocardial infarction, improving IK1 remodeling in rats (Li et al., 2015). H2S-mediated Sp1 S-sulfhydration significantly contributes to cardiovascular disease prevention and treatment. H_2_S restores Sp1-CSE-H_2_S pathway activity by enhancing Sp1 S-sulfhydration, promoting homocysteine metabolism, and alleviating hyperhomocysteinemia. Additionally, restoring the H_2_S/Sp1/VEGFR-2 pathway addresses endothelial dysfunction caused by CBS deficiency, while the H_2_S/Sp1/KLF5 pathway inhibits Ang II-triggered cardiac hypertrophy. The H_2_S/Sp1/MMP2 pathway regulates elastin activity, treating aortic aneurysms caused by permanent aortic wall dilation (Zhu et al., 2022).

In developing drugs for cardiovascular diseases, several key points should be considered. First, since Sp1 phosphorylation is involved in many cardiovascular diseases through various pathways and functions, targeting Sp1 phosphorylation can be a crucial direction for drug development. Additionally, oxidation, a novel type of PTM associated with cardiomyopathy, warrants attention (Roman et al., 2020). Examining comprehensive modification sites is essential for developing targeted drugs. High-frequency modification sites such as Thr453, Thr739, Thr668, Ser670, Cys664, and Lys703 should be prioritized. Targeting these sites may significantly impact the treatment of various cardiovascular diseases. Many natural compounds extracted from plants, such as luteolin, kaempferol, and cyanidin-3-glucoside (Cy3G), belong to flavonoids, which play a pivotal role in preventing cardiovascular diseases due to their anti-thrombotic, anti-inflammatory, anti-atherosclerotic, and antioxidant properties (Ciumărnean et al., 2020). Studies in both cells and animals have confirmed that these compounds positively affect gene expression by modulating Sp1 PTMs. These compounds influence the expression of specific genes and ultimately treat cardiovascular disorders by controlling these modifications. However, additional investigation is required to elucidate the molecular pathways. The complexity of Sp1 functions necessitates considering the synergistic effects between various PTMs in regulating disease-linked genes. Moreover, DNA methylation of Sp1 at its promoter binding sites, in addition to these histone post-translational changes, is also crucial. These findings provide fresh perspectives on the role of Sp1 in cardiovascular disorders.

Overall, Sp1 PTMs are essential in the development and advancement of various cardiovascular diseases. Studying Sp1 PTMs not only enhances our understanding of disease pathogenesis but also aids in developing new drug targets and biomarkers. This research is of great significance for the prevention and treatment of cardiovascular diseases.
